# Report on an international workshop on kangaroo mother care: lessons learned and a vision for the future

**DOI:** 10.1186/s12884-018-1819-9

**Published:** 2018-05-16

**Authors:** Adriano Cattaneo, Adidja Amani, Nathalie Charpak, Socorro De Leon-Mendoza, Sarah Moxon, Somashekhar Nimbalkar, Giorgio Tamburlini, Julieta Villegas, Anne-Marie Bergh

**Affiliations:** 1 Clinical Epidemiology and Public Health Research Unit, Institute for Maternal and Child Health-IRCCS Burlo Garofolo, Trieste, Italy; 2 Child and Newborn Health, Ministry of Public Health, Yaoundé, Cameroon; 3 Kangaroo Foundation, Bogotá, Colombia; 4 Kangaroo Mother Care Foundation, Manila, Philippines; 5 Maternal, Adolescent, Reproductive and Child Health Centre, London School of Hygiene and Tropical Medicine, London, UK; 6 Department of Pediatrics, Pramukhswami Medical College, Karasmad, India; 7 Center for Child Health and Development, Trieste, Italy; 8 SAMRC Research Unit for Maternal and Infant Health Care Strategies, University of Pretoria, Pretoria, South Africa

**Keywords:** Prematurity, Kangaroo mother care, Implementation, Scale-up, Training, Quality of care

## Abstract

**Background:**

Globally, complications of prematurity are the leading cause of death in children under five. Preterm infants who survive their first month of life are at greater risk for various diseases and impairments in infancy, childhood and later life, representing a heavy social and economic burden for families, communities and health and social systems. Kangaroo mother care (KMC) is recommended as a beneficial and effective intervention for improving short- and long-term preterm birth outcomes in low- and high-income settings. Nevertheless, KMC is not as widely used as it should be. The International Network on KMC runs biennial workshops and congresses to help improve the coverage and quality of KMC worldwide. This paper reports the results of the two-day workshop held in November 2016, where 92 participants from 33 countries shared experiences in a series of round tables, group work sessions and plenaries.

**Findings:**

Barriers to and enablers of KMC are discussed with regard to parents, health workers and the health system. Key factors for effective implementation and uptake relate to appropriate training for health staff, adherence to protocols and the creation of a welcoming environment for families. Recommendations for planning for national programmes are made according to a six-stage change model. Resources and the cost of making progress are discussed in terms of investment, maintenance, and acceleration and scaling-up costs. KMC training requirements are presented according to three levels of care. To ensure quality KMC, key requisites are proposed for the different KMC components and for sensitive communication with caregivers. The group attending to the monitoring and evaluation of KMC at a national and subnational level highlight the lack of standard indicator definitions. Key priorities for investment include health services research, harmonisation of indicators, development of a costing tool, programming and scaling up, and the follow-up of preterm infants.

**Conclusion:**

It is hoped that this report will help to further scale-up and sustain KMC through a systematic approach that includes raising commitment, identifying key strategies to address the main barriers and using existing facilitators, ensuring training and quality, agreeing on indicators for monitoring and evaluation, and advancing implementation research.

## Background

Globally, complications of prematurity are the leading cause of death in children under five (18%), surpassing pneumonia (16%) and diarrhoea (9%). The majority of premature deaths (16%) occur in the neonatal period (first four weeks of life), with about two-thirds occurring as early neonatal deaths in the first week of life. Within this first week, most of the deaths occur during the first 48 h of life. In absolute terms, about two-thirds of the estimated 2.7 million annual neonatal deaths in the world occur in preterm infants, mostly in South Asia and sub-Saharan Africa [1, 2]. The risk of death in preterm infants is inversely associated with gestational age and is higher in preterm infants that are also born small for gestational age [3]. Within countries, the risk of death is also higher in infants from poorer households [1].

Neonatal and child deaths are not the only consequence of prematurity. Of the estimated 13 million preterm infants who survive their first month of life, almost 3 % suffer from moderate or severe long-term neurodevelopmental (cognitive and behavioural) impairment and about four-and-a-half per cent suffer from mild impairment. In addition, globally almost 200,000 preterm infants are affected by retinopathy of prematurity, with 32,000 having visual impairment, including blindness [4, 5]. Other important conditions more frequent in preterm than in full-term infants include infections and jaundice that can lead to hearing impairment [6], and feeding difficulties and malnutrition, which often causes stunting [7]. Prematurity, especially when associated with intrauterine growth restriction followed by rapid post-neonatal weight gain, increases the risk of non-communicable diseases (high blood pressure, cardiovascular diseases, obesity, diabetes) later in life [8]. Finally, all the above contribute to major negative social effects, as measured by decreased educational attainment, employment and economic productivity for individuals, and by an increased social and economic burden for families, communities and health and social systems [9].

Kangaroo mother care (KMC) is recommended as a beneficial and effective intervention for improving preterm birth outcomes [10–12]. Compared with conventional care, KMC can reduce the following: mortality at discharge and at 40 to 41 weeks’ postmenstrual age or at latest follow-up; severe and nosocomial infection/sepsis; severe illness and lower respiratory tract disease; hypothermia; hypoglycaemia; and hospital readmissions. Moreover, KMC increases weight, length, and head circumference gain, breastfeeding at discharge or at 40 to 41 weeks’ postmenstrual age and at one to three months’ follow-up, maternal satisfaction and maternal-infant attachment, and home environment [13–15]. KMC also has positive short- and long-term neurodevelopmental, cognitive, emotional, behavioural and social effects [16–19].

The International Network on KMC (INK) was created in 1996 in Trieste, Italy, during the first International Workshop on KMC [20, 21]. Its objectives included advocacy and support for KMC projects and programmes, as well as dissemination and sharing of information among researchers and health professionals. The latter were achieved through the organisation of biennial workshops and congresses. The last event, marking the twentieth anniversary of INK, was held in Trieste in November 2016. Invitations were disseminated through the established channels of communication and attendance was open to any interested person.

The two-day congress hosted over 100 oral and poster presentations on experiences in 30 countries in all parts of the world. There were 180 participants from 38 countries, including 101 doctors (mostly paediatricians and neonatologists), 23 nurses, 5 midwives, a number of nutritionists, psychologists, physiotherapists, programme managers, and two mothers. All of them had practical experience in KMC as researchers, practitioners or managers. The congress was preceded by a two-day workshop attended by a smaller group of 92 participants from 33 countries,^1^ with a similar breakdown by profession. The two events did not have an official sponsor. Travel, accommodation and registration expenses of the majority of participants, including speakers and workshop facilitators, were self-funded or funded by their own institution. One invited speaker did not receive authorisation for attending and another fell ill.

The objective of the workshop reported on in this paper was to gather and discuss experiences of KMC programmes at different levels of health systems and to provide ideas for future improvements. The programme included round tables, group work and plenaries on the following: barriers to and enablers of KMC; key factors for effective implementation and uptake; planning for national programmes; resources and the cost of progress; training; ensuring quality; monitoring and evaluation; and key priorities for investment. In each workshop session, an appointed secretary took notes and wrote a summary, in collaboration with an appointed coordinator. Summaries were discussed and modified as needed during plenary sessions. These data sets formed the basis for this paper reporting on the outcomes of the deliberations on the key themes highlighted in the different working groups.

## Barriers to and enablers of kangaroo mother care

Overcoming barriers to the practice of KMC and harnessing enablers can assist in synchronising effective KMC implementation. Workshop participants shared their own experiences on many of the barriers and enablers that have also been reported in the literature and categorised them in terms of what they entail for parents, health workers and the health system.

### Barriers

Participants mentioned perceived barriers which make it difficult for *parents* to practise KMC and which are similar to experiential, resource-related and sociocultural barriers identified in the literature [22]. Experiential barriers include fear of hurting the infant, pain/fatigue during KMC, the difficulty of adhering to the kangaroo position while sleeping or in high temperatures, concerns over breast milk expression, feeling forced to take on KMC, lack of support from and negative attitudes on the part of staff. These experiences can be further complicated by resource-related barriers such as a lack of knowledge of KMC, difficulty in accessing the health facility, deprived hospital environments and shortage of rooming-in facilities for parents. Culture-specific beliefs and disapproval by the community appear to play a lesser role. Concerns related to gender roles included fathers reporting a lack of opportunity to practise KMC.

The most common *health-worker* barriers [22, 23] for nurses mentioned by participants were the absence of clear guidelines and quality training, a workload increase when not properly trained, reduced resources and support, doubts about the efficacy and benefits of KMC, and concerns related to patients’ medical conditions. For doctors, the prime barriers highlighted were scepticism about KMC efficacy, lack of understanding and acceptance of KMC, and insufficient training. Further barriers include the absence of post-training evaluation and monitoring, non-coverage of KMC in university curricula, and challenges with record keeping.

At *health systems* level [23, 24], identified barriers include absence of leadership (leading to infrastructure shortages, lack of space and poor privacy in hospitals), lack of provision of transportation services to health facilities, inflexible visitation policies for parents, insufficient staff and leaders in the field, and an absence of KMC champions (e.g. in rural western India [25]). Perverse financial mechanisms that rewarded traditional incubator/warmer care instead of promoting prolonged skin-to-skin contact were also discussed. Reference was made, for example, to a minor drop in KMC practice (7.7%) after paediatricians in Gujarat, India, had acquired a warming device [26].

### Enablers

For *parents* [22], the principal enablers were considered to be social support from family members, friends, other mothers and staff, along with mother-infant bonding and an increase in mothers’ sense of confidence and empowerment. Powerful enablers are an understanding of the efficacy of KMC, leading to it being preferred to traditional care, and the belief that infants enjoy KMC. The possibility of remaining with the infant in hospital and access to KMC knowledge are further enablers.

Compelling enablers among *nurses* [27] include the experience and commitment of senior nurses, exposure to pre-service KMC training and observing the benefits of KMC in practice. Where nurses have experienced a decrease in workload, there also appears to be increased support for KMC.

In the *health system* [27, 28], commitment from hospital directors and senior staff can facilitate the allocation of space, staff and equipment and promote an enabling environment to support KMC. Other KMC enablers include quiet, private rooms and separator screens, access to a human milk bank, good communication across the health system, and committees that advocate KMC. Establishing KMC training centres of excellence (CoEs) and creating a KMC network among health facilities and professionals can support national diffusion and scale-up. Not only are KMC data collection and entry important, but so are the sharing of results and best practices with hospital directors and staff. A national KMC policy (with periodic updates) allows for public-sector resource allocation and standards setting in the private sector.

## Key factors for effective implementation and uptake

Mechanisms for effective implementation and uptake are closely related to the barriers and enablers discussed in the previous section. Workshop participants agreed that the availability of trained and dedicated KMC staff and tools to provide KMC education and practise quality KMC are key factors in implementation. Parental acceptance of KMC seldom appears to be an issue if appropriate counselling is provided. A welcoming, open environment for families with provision for mothers, such as comfortable chairs, supporting binders and access to food and water, helps families to sustain KMC.

Training all staff to follow a KMC protocol that includes eligibility criteria for both the neonatal unit and the KMC ward helps avoid inconsistent implementation. Ensuring that mothers and infants return for follow-up is challenging, especially after the 40-week appointment, which is necessary to evaluate the infant’s progress. mHealth technologies to remind mothers or caregivers of appointments can help minimise this challenge [29]. A key factor to consider across the trajectory of KMC from initiation to follow-up and ambulatory KMC is a proper budget for KMC services. Parents’ out-of-pocket expenses should also be taken into account [23, 30].

## Planning for national programmes

The working group on planning described the need to embed a national KMC programme in government strategic planning for maternal and newborn care across all levels of the health system and in all the health systems building blocks. A national programme should also have the following features: broad stakeholder participation; availability of implementation guidelines (including standards and protocols); minimum criteria set for KMC services; and the existence of various essential planning activities and documents (policy, operational, financing and resource allocation, monitoring and evaluation). The emphasis placed on the different KMC programme features may vary from one country to another.

A stages-of-change model specifically developed for the monitoring of progress with the implementation of KMC was used to identify the factors and actions that should ideally be taken into account at each stage of implementation. The model has three phases and six stages: create awareness and commit to implement (pre-implementation phase); prepare to implement and implement (implementation phase); integrate into routine practice and sustain new practices (institutionalisation phase). The process of planning and implementation is cyclical and the movement between the different stages may be different for different activities [31, 32]. Table 1 contains a summary of important planning factors identified by the group.

**Table 1:** Planning for a national KMC programme using a stages-of-change approach

Stage	Important factors to consider
1. Create awareness	• Leaders and champions at different levels (global, national, regional) • Use of best available, applicable evidence • Advocacy by key government officials and others • Key stakeholders for potential funding and other contributions • Exploring policy formulation and integration of KMC into different state programmes
2. Commit to implement	• Leadership and government commitment in the context of a team approach • Technical working group • Government guidelines; begin drafting standard operating procedures/protocols • Potential resources • Draft operational plan • Accountability measures for different stakeholders
3. Prepare to implement	• Needs assessment and cost-benefit analysis • Targets formulated • Policies, operational plans and protocols refined • Budgets drafted and resources allocated (government and donors) – accountable tracking of allocations included • Selection of key staff to be trained and scale-up of training in a manageable way (often integrated into other training programmes, e.g. Essential Newborn Care) • Centre(s) of excellence (CoE) identified, strengthened or established – systematic linkages between CoEs and district/regional health facilities ensured • Monitoring and evaluation system with key indicators to allow for learning from processes – regular feedback and mid-course corrections
4. Implement	• KMC team empowerment • Continued advocacy and work with media • Programme integrated into state and central budgets (a ‘budget line’) • Donor and insurance agencies and companies on board • System for information, education and communication (IEC) established and materials developed • Number of CoEs expanded • Conducive environment created at health facility level (e.g. KMC culture, peer group work, in-service training)
5. Integrate into routine practice	• Continuation of leadership and advocacy • Continued financial support from government and international community • Monitoring framework established (preferably electronic) (capacity for collecting, analysing and using data) • Clinical outcomes measured at facility and health system level • Further evidence generated through research (at CoE and district level) • Integration with institutional quality assurance and quality improvement • Well-organised follow-up system for growth monitoring and promotion of neurodevelopment • Expansion of parent education opportunities
6. Sustain new practices	• Monitoring of national progress • Continuous policy review • Budgetary support (including insurance) sustained • Accountability teams overseeing monitoring and feedback • Continued training and refreshers • Newly generated evidence disseminated at national conferences • KMC integrated into pre- and in-service education • Champions nurtured and new ones identified

## Resources and costs: What is needed to make progress?

Implementing a KMC programme is not without cost. While the conceptual framework is built around the mother’s care for her small baby, human and technical resources are nevertheless required to enable the parents to learn, adapt, and become confident in providing KMC to their fragile baby. This should start in the hospital, under the supervision of healthcare personnel, and continue at home, when the parents are on their own. Taken to a higher level, similar resources on a larger scale are required for KMC to be adopted by countries as the standard of care for all low birth weight (LBW) infants.

Rates of remuneration for health personnel and capital outlay for a KMC unit vary widely from country to country. Therefore, instead of producing estimates for personnel costs and capital outlay, the working group generated an inventory of resources essential for establishing, sustaining and accelerating KMC at facility, subnational and national levels. Excluding what is expected to be available as part of routine care for every preterm and/or small baby, cost and resource requirements for KMC were classified into three categories: investment costs; maintenance costs; and acceleration and scaling-up costs.

*Investment costs* include those that relate to training and capacity building of healthcare personnel, as well as to establishing a KMC centre of excellence. Components of training to which costs are attached include preparatory meetings, training of trainers or resourcing from an existing pool of trainers, the production of materials or kits, the training itself (e.g. venue, meals, accommodation), and the evaluation of training.

A KMC facility could be established simply to provide services to its clients or to serve as a centre of training and excellence for other facilities in the region or country. Regardless of this goal, the basic KMC unit requires the following:

- space for beds and/or armchairs for extended stays by KMC dyads or families;
- facilities and supplies for water, sanitation, and hygiene;
- environmental modifications to facilitate transition to home;
- nurse or midwife station for documentation; and
- equipment to facilitate and support breastfeeding.

As an adjunct to this, strategies on fundraising and ways to approach potential funders may be needed to initiate and establish the first KMC project or programme. There are global initiatives and financing facilities that can provide support for this endeavour. Global development partners in health are accessible through various mechanisms if a direct approach is not possible.

*Maintenance costs* include the cost of

- supervision, coaching, and (re)training;
- advocacy campaigns and fund-raising (if needed); and
- continuous quality improvement (QI) activities.

Maintaining quality KMC service provision relies heavily on staff retraining and coaching with the aid of the facility’s team of trained trainers or resources from the regional centre of training and excellence. Advocacy campaigns include those that are intended to raise awareness and generate demand through context-specific and culture-specific information, education and communication (IEC) materials, small group discussions, or inclusion of KMC in a family-centred care kiosk if available in the facility. Budget allocation for continuous QI implies the creation of or inclusion of KMC in a data retrieval system that will enable continuous evaluation of the KMC programme and its overall impact on maternal and neonatal outcomes. The same information may stimulate the undertaking of operational or clinical research that could impact on local policy and quality practice of KMC.

To sustain KMC programmes, steps must be taken to integrate KMC into the national health system, including in the areas of budget planning and allocation. Ancillary to this, KMC would have to be included in health insurance systems (government and private), for which the assistance of an actuarial expert might be necessary.

*Acceleration and scaling-up costs* involve partnerships with government and development partners until it is clear that the technology has been adopted on a national scale. KMC in pre-service and in-service training requires collaboration with academia, professional societies and other training centres for healthcare professions. Regulatory commissions providing continuing medical education or continuous professional development credits for KMC post-graduate training may also be an avenue for generating more buy-in to KMC as a learned technology. The costs related to implementation and evaluation research and publication are crucial if collaboration in the scale-up phase of KMC is to be increased.

## Training

The provision of high-quality and effective KMC training by experienced and senior trainers is one of the major challenges in accelerating worldwide dissemination of KMC. Health systems provide different levels of care, each requiring different levels of knowledge and skills; this is also applicable to KMC. The objective of the deliberations of the working group on training was to systematise training requirements according to the level of care and to propose indicators to evaluate the effectiveness of KMC training and the functioning of newly trained KMC centres. Different countries classify their levels of health care differently and the description in Table 2 should merely be regarded as a broad guideline. Some of the training guidelines included do not pertain exclusively to KMC, but to the care of sick and small newborns in general.

**Table 2:** KMC training needs for different levels of care

Level	Description	KMC training focus	Indicators for effective KMC training
1	Primary health care in the community • Births occur at community health centres or at home with the assistance of traditional birth attendants or community workers • All LBW and premature infants must be referred to a higher level of care for evaluation by a doctor or paediatrician	Use this opportunity to integrate essential newborn care and basic concepts of resuscitation into one package. • Transportation: - Identification of pregnant women at risk of premature delivery and in utero transfer before infant is born - Infants not referred before birth: how to avoid hypothermia with the kangaroo position during the transfer of the infant • Initiating kangaroo nutrition as soon as possible (breastmilk or water with sugar if mother is not available) to avoid hypoglycaemia • Sensitisation of the community regarding KMC • Teaching mothers and families how to practise KMC and detect danger signs • Familiarity with the most common problems and risk factors for all small and premature babies once discharged, and awareness of the need to ensure both medical follow-up and support to the family in a higher level of care or in a KMC program​me	• Mortality • Number of infants referred in kangaroo position • Number of LBW and premature infants who received breastfeeding within the first hour after birth
2	Corresponds to district, provincial or regional hospitals with or without neonatal units and with health staff varying accordingly	*All healthcare workers:* • Use of simple tools such as the Ballard test for assessment of gestational age *Hospital setting without a neonatal unit:* • Providing the initial management of immediate complications before referring the infant in the KMC position to a facility with a neonatal unit *Hospital setting with a neonatal unit and ability to manage complications of prematurity (*e.g. *jaundice, sepsis, respiratory distress):* • Full KMC training: - See also level 3 - Set-up and running of a KMC unit/ward - Conducting KMC follow-up clinics, with neurological assessment (could be high-risk follow-up, depending on availability of trained staff and the number of infants attended in the institution)	• Mortality (including cause of death) • Length of hospital stay • Breastfeeding rates • Number of days the infants received KMC • Infection rates during admission • Number of LBW and premature infants who arrive from level 1 with hypothermia or hypoglycaemia
3	Corresponds to teaching and university hospitals with neonatal intensive care (such as advanced ventilation and nutritional support)	*Full KMC training* • KMC components: - kangaroo position - kangaroo nutrition - early discharge with strict follow-up in the KMC ambulatory programme • Establishment and running of a KMC ward • KMC data collection and analysis • Initiation of KMC: should start as soon as possible, ideally in the delivery room or neonatal intensive care unit (NICU) • Transfer to level 2: once infants have been treated for complications they can be transferred back (‘stepped down’) to level 2 after completing 40 weeks of gestational age (presumed term) • High-risk follow-up to at least one year of corrected age: - monitoring of somatic growth and neurosensory and neuro-psychomotor development - with timely intervention by physical, audiometric or optometric therapists when needed • Specific developmental problems and needs of premature babies, including psychosocial support to parents (verbal and written information for and counselling of parents to improve their ability to cope with the new situation at home).	• Same as level 2 • Nutrition at discharge, 3, 6, 9 and 12 months corrected age • Neuro-psychomotor and sensory development • Number of infants readmitted to the NICU or KMC ward

## Ensuring quality

Although it is essential to establish KMC units or wards in hospitals providing care to premature and LBW infants and ensure that the highest proportion of eligible babies have access to KMC, this is by no means enough to guarantee that appropriate KMC care is provided. A focus on quality of care and QI initiatives using the QI cycle is, however, rare. Neonatal intensive care and KMC unit personnel providing care to small babies and their caregivers should adhere to quality standards so that the interventions are effective. The World Health Organization recently developed a comprehensive set of maternal and newborn care standards, which include components associated with KMC [33]. However, the standard remains generic and it does not provide quality criteria for KMC. Key requisites for effective KMC include:

- skin-to-skin contact as soon as possible after birth and for as long as possible each day (preferably 18 h or more);
- breastfeeding or breastmilk administered by naso- or orogastric tubes or cup feeding when necessary (where infant and mother are capable); and
- context-appropriate discharge criteria and follow-up provided to the baby and his/her family.

Additional important quality requisites should include the following: clear, complete and culturally sensitive information provided to caregivers during their stay in the health facility and at discharge; a friendly and empowering attitude on the part of health professionals; adequate links with health providers in charge of follow-up; and other community resources to support the family after discharge, which is a very demanding period for parents.

In many countries, the quality of care of LBW and premature infants is included in assessments of the quality of maternal and newborn care – in some countries systematically and in others anecdotally. Although assessment and improvement of the quality of KMC should ideally not be done in isolation, but as part of initiatives addressing the comprehensive care of all babies, it is not always known to what extent KMC and its components are included in these evaluations and in subsequent QI actions.

## Monitoring and evaluation of kangaroo mother care at a national and subnational level

KMC currently lacks the standard indicator definitions that are used at a national and subnational level for monitoring and evaluation. As with many newborn interventions, data on KMC coverage and process are sparse and are not routinely available in many low- and middle-income countries, making tracking of national KMC programme scale-up especially challenging [2, 34].

There are a number of reasons for the lack of data on KMC. Firstly, the intervention involves a number of components, including continuous and prolonged skin-to-skin contact (between mother and newborn), kangaroo nutrition and early discharge [35, 36]. Apart from clinical care charts, these components are difficult to capture accurately through routine health information systems. The WHO recommends KMC for babies weighing < 2000 g [36], but many national programmes recommend KMC for all LBW babies (< 2500 g). In some settings, there is confusion between skin-to-skin care at birth (for all babies) [23] and KMC (for LBW, predominantly preterm newborns). Such factors make the intervention difficult to compare between settings. Capturing the population in need (to measure an appropriate population level denominator) is particularly challenging for KMC [2], especially where countries may have limited capacity to identify and record LBW [37] and assess gestational age. For example, in some settings fewer than 50% of babies are weighed at birth and even fewer have access to first trimester ultrasound for accurate gestational age assessment [38]. Finally, maternal reporting of KMC is not currently carried out by routine household survey platforms due to difficulties with the sample size and the achievement of sufficient sampling power through a representative national survey.

Some middle-income countries, especially in Latin America, have detailed programme data on KMC, but there is no existing standardised coverage indicator definition for KMC. In the absence of coverage data at a national and subnational level, process indicators (indicators of programme performance) may be more measurable and more useful in existing data systems.

The discussion of the monitoring and evaluation working group was based on a monitoring framework developed in conjunction with the Kangaroo Acceleration Partnership and the Every Newborn Action Plan at the previous KMC conference in Kigali, Rwanda, in 2014. The framework was developed through literature review and face-to-face consultations with multiple partners and KMC experts working at country and global levels. The resulting measurement framework includes 10 indicators based on two main components:

- service readiness, based on the WHO building blocks framework; and
- service delivery action sequence covering identification, service initiation, continuation to discharge, and follow-up to graduation [39].

The group discussed the different areas of the KMC action sequence framework and compared the capacity of the 12 middle- and high-income countries represented in the group to collect this data within their existing national and subnational level monitoring systems. The countries were Brazil, India, Indonesia, Iran, Colombia, Nicaragua, Vietnam, the Philippines, Canada, Italy, Norway and Sweden. Seven of the countries recorded the initiation of KMC services at the facility level and would be able to aggregate this data at a national level (as a proxy for KMC coverage). Only six countries had the capacity at national level to identify the numbers of LBW babies, and their gestational age. Less than half of the countries could collate data on the continuation of KMC until discharge, while seven countries collected data on the follow-up of KMC babies after discharge. Although this exercise was limited to a small number of higher- and middle-income settings, the discussion provided important guidance on the need and demand for standardised monitoring systems that track the care of small and sick newborns, including KMC. Monitoring and evaluation of KMC must be integrated into national monitoring systems, alongside other newborn care indicators. Most participants expressed the need for standard quality indicators (on structures and processes at the facility level), as well as key outcome indicators. Health facilities should also develop context-specific QI processes based on existing tools and suited to institutional systems.

The use of a core indicator framework to guide implementation and scale-up could help strengthen KMC programmes, increase coverage and facilitate global tracking of KMC implementation. As data collection systems advance, programme managers and evaluators should be encouraged to document their experiences using this framework to measure progress and allow indicator refinement, with the overall aim of working towards sustainable, country-led data systems.

## Key priorities for investments

The process of identifying priorities within investment alternatives to KMC takes costs and impacts into consideration. Working group participants agreed that individual countries should determine their own investment priorities, as they are at different levels of development and implementation regarding KMC. Investments should aim to release the vast potential of KMC, and the key challenge is to prioritise investments that will most advance KMC in the country. Five main areas of priority investments were identified: research; harmonisation of indicators; development of a costing tool; programming and scaling up; and follow-up.

*Investment in research*. The key issue in the realisation of KMC programmes is to find robust context-specific evidence supporting the efficiency and efficacy of KMC. The need to invest in research is vital to demonstrate the value and benefits of KMC, not only for improving health but also for promoting child development.

*Harmonisation of indicators*. To be sustainable, harmonised KMC indicators need to be built into national health information systems, data collection, monitoring (including quality, audit), and evaluation.

*Investing in a KMC costing tool*. Developing a KMC costing tool is critical in facilitating budgeting. As such, the KMC costing tool should serve as a reference tool that will guide organisations to streamline budgeting processes by ensuring that all costs are calculated at the outset, with a clear and accurate picture of the financial implications of the various components.

*Investment in KMC programming and scaling-up*. Workshop participants agreed that KMC had not reached premature babies to the required extent, so there is a need to change the pace of scale-up. An investment in innovative approaches to service delivery is needed, with careful consideration of quality, and attention to the implementation barriers. Implementation should include appropriate care for premature and LBW babies from birth, including planning for an appropriate place of birth for premature deliveries below 33 weeks of gestational age.

Participants also agreed that it was important to engage the private sector in implementation efforts, because it delivers a significant proportion of maternity services in many countries. The private sector could also be engaged in financing mechanisms. In Cameroon, for example, a development impact bond is being set up that would use private capital to provide the initial financing to scale up KMC, with investors being paid back at varying rates, depending on their country’s performance in rolling out KMC [40].

*Follow-up*. In order to maximise the benefits of KMC, it should be complemented by a comprehensive, longitudinal follow-up starting from discharge. Discharge should be accompanied by adequate information to mothers and family members and the provision of links to primary health care and the community.

## Conclusion

The International Workshop and Congress on KMC showcased the progress made in the implementation of KMC in healthcare institutions and the increase in coverage of services. The overwhelming atmosphere among participants was one of passion, commitment and dedication to the intervention and an appreciation of the importance of sharing experiences and learning from each other. However, a number of challenges were also highlighted. For sustainability and further scale-up of KMC, a systematic, comprehensive approach is required which should include:

- engaging more governments to implement KMC at scale;
- ensuring a sustained commitment to action among established implementers;
- identifying key strategies to address the main barriers and using existing facilitators or exploring innovative ones;
- ensuring quality training;
- promoting quality improvement; and
- agreeing on indicators that would truly reflect the impact of KMC on health and development of the preterm and LBW population.

The workshop tried to advance the acceleration agenda by putting forward concrete, practical actions, complemented by rigorous scientific research, programme implementation, and monitoring and evaluation.

## Abbreviations

CoE: Centre of excellence
IEC: Information, education and communication
INK: International Network on KMC
KMC: Kangaroo mother care
LBW: Low birth weight
NICU: Neonatal intensive care unit
QI: Quality improvement

## Footnotes

1 Bhutan, Canada, Congo Republic, Ethiopia and Zimbabwe were not represented at the workshop. The following countries were represented at both the congress and the workshop: Algeria, Bangladesh, Belgium, Brazil, Cameroon, Colombia, Finland, France, India, Indonesia, Iran, Israel, Italy, Madagascar, Malawi, Nicaragua, Nigeria, Norway, Philippines, Poland, Rwanda, Slovenia, South Africa, Spain, Sweden, Switzerland, Tanzania, Turkey, Uganda, Ukraine, United Kingdom, United States, Vietnam.
